# Evaluating Public Awareness and Understanding of Anesthesia Practices in Saudi Arabia: A Cross-Sectional Study on Patients’ Confidence in and Perceptions of Anesthesiologists

**DOI:** 10.7759/cureus.102374

**Published:** 2026-01-27

**Authors:** Amani Nabri, Zaid A Alaboudi, Noor I Al Dhaif, Khalid S Alshalawi, Deema A Aljasser, Aljouri A Alrazoog, Fayez K Alanazi, Sara A Alshaikh, Mohammed F Alanazi, Abdulmohsen F Alanazi, Zaid A Alaboudi

**Affiliations:** 1 College of Medicine, Dar Al Uloom University, Riyadh, SAU; 2 Clinical Sciences, College of Medicine, Dar Al Uloom University, Riyadh, SAU; 3 Family Medicine, Dar Al Uloom University, Riyadh, SAU; 4 Medicine and Surgery, Dar Al Uloom University, Riyadh, SAU; 5 Clinical Sciences, Dar Al Uloom University, Riyadh, SAU; 6 Emergency Medical Services, Saudi Red Crescent Authority, Riyadh, SAU; 7 Radiology, Imam Abdulrahman Al-Faisal Hospital, Riyadh, SAU

**Keywords:** anesthesia awareness, anesthesiologist role, informed consent, patient perception, public knowledge

## Abstract

Background: Adequate public awareness of anesthesia practices and the role of anesthesiologists is essential for informed consent, patient confidence, and perioperative safety. Despite advances in anesthetic care, gaps in patient knowledge and misconceptions remain common.

Methodology: This cross-sectional study was conducted among adults aged 18 years or older with a prior history of elective anesthesia. Data were collected using a structured, self-administered online questionnaire distributed via Google Forms. The survey assessed demographic characteristics, prior anesthesia exposure, knowledge of anesthesia types, perceptions of anesthesiologists’ roles, fears, communication quality, trust, and satisfaction.

Results: A total of 298 participants were included, of whom 155 (52.0%) were female. General anesthesia was the most frequently reported type (190, 63.8%). Awareness of general anesthesia (272, 91.3%) and local anesthesia (263, 88.3%) was high, but knowledge of regional techniques was limited, including spinal anesthesia (47, 15.8%), epidural anesthesia (39, 13.1%), and peripheral nerve blocks (45, 15.1%). Familiarity with the anesthesiologist’s role was reported by 239 participants (80.2%), and 212 (71.1%) correctly identified that the anesthesiologist remains in the operating room throughout surgery. The most common anesthesia-related fear was experiencing pain during the procedure (138, 46.3%). Communication outcomes were favorable, with 106 participants (35.6%) receiving complete explanations and 268 (89.9%) reporting either being satisfied or very satisfied. Female participants demonstrated significantly higher awareness scores than males (*P* < 0.001). Being informed about anesthesia consent (208, 69.8%) and preferring to meet the anesthesiologist preoperatively (208, 69.8%) were associated with significantly higher awareness and perception scores (both *P* < 0.001). Regression analysis identified preference to meet the anesthesiologist as the strongest positive predictor of both awareness and perception while receiving regional anesthesia negatively predicted perception scores.

Conclusions: Although patient trust and satisfaction with anesthesiologists were high, significant gaps remained in knowledge of regional anesthesia. Preoperative education regarding anesthesia can enhance public awareness and perceptions of anesthesia care.

## Introduction

Over time, anesthesiology has evolved into a critical medical specialty involved in perioperative care. Since its inception in the 19th century, anesthesiology has expanded from merely enabling surgery to a comprehensive field including pain management, critical care, palliative care, and emergency medicine [1]. Modern anesthesiologists not only administer anesthetic agents but also play key roles in intensive care units (ICUs) and postoperative pain services [2,3]. These expanding responsibilities make anesthesiologists essential contributors to patient safety and comfort throughout the surgical experience. Therefore, public understanding of anesthesia practice is crucial [4]. Despite this evolution, several studies have reported that patients often lack a clear understanding of anesthesia and anesthesiologists. For instance, a study from Qassim province found that even patients with prior surgical experiences had limited knowledge about anesthesia [5]. At King Khalid University Hospital in Riyadh, only a small fraction of participants recognized anesthesiologists as specialized doctors, with even fewer aware of their critical role during surgery. These gaps were notably larger among less educated and lower-income groups [6].

This gap in public awareness has practical implications. Patients who misunderstand anesthesia may experience undue anxiety or fear of rare complications such as awareness under anesthesia, which can hinder effective communication and cooperation during perioperative care. In fact, studies have linked poor anesthesia knowledge and misconceptions to increased preoperative anxiety, prolonged recovery times, and other adverse outcomes [7]. Conversely, patient education and engagement have been shown to improve satisfaction and safety. For instance, enhancing preoperative communication and education about anesthetic procedures has been associated with higher patient trust and satisfaction [8]. However, data from Saudi Arabia also highlights a knowledge deficit among the general public. A study involving adults residing in KSA concluded that a significant positive correlation between perception and knowledge scores was found. Higher perception scores were associated with having a chronic medical condition, while higher knowledge scores were associated with being female and having undergone more surgeries. Anesthesiologists were recognized as specially trained doctors by 79.8% of participants, and 63.8% trusted physicians for care [8]. Similar findings are echoed globally. In Ethiopia, only about 28.3% of participants had a satisfactory level of knowledge regarding anesthesia and anesthetists [4]. In South Korea, a national survey revealed that 25.2% of the population was unaware that anesthesiologists manage anesthesia during surgery, and most lacked an understanding of their broader roles [9].

Similarly, another study found that 90% of patients were unaware of anesthesia complications, and nearly half did not recognize that an anesthesiologist is a doctor. These findings highlight the critical need for continuous educational efforts within healthcare settings [10]. Furthermore, a survey revealed that while most patients knew that anesthesiologists were medical doctors, only a minority understood the extensive training required or the diverse roles anesthesiologists play beyond the operating room, such as managing labor pain and critical care [11]. Therefore, the present study aimed to assess public awareness and understanding of anesthesia practices and the role of anesthesiologists among adults with prior exposure to elective anesthesia. The primary objective was to evaluate participants’ knowledge of different anesthesia types and their understanding of anesthesiologists’ roles during perioperative care. Secondary objectives included assessing patients’ perceptions, fears, confidence, and satisfaction related to anesthesia, as well as identifying demographic and clinical factors associated with variations in awareness and perception scores.

## Materials and methods

Methodology

Study Design and Study Setting

This cross-sectional study was conducted to assess awareness, perceptions, and experiences related to anesthesia and the role of anesthesiologists. The study was conducted using an online and mobile-based platform through Google Forms, targeting adults in the community with a prior history of elective anesthesia.

Study Participants

The study population included adult individuals aged 18 years and above who had previously undergone any form of elective anesthesia, including general, local, or regional anesthesia such as spinal, epidural, or peripheral nerve blocks. Participants were recruited from the general community and represented diverse demographic characteristics, including age, gender, education level, and income. Individuals younger than 18 years, critically ill patients, and those unable to provide informed consent were excluded.

Sample Size and Sampling Techniques

The required sample size was calculated assuming a 95% confidence level (*Z* = 1.96), a margin of error of 5% (*E* = 0.05), and an estimated population proportion of 0.5 to maximize sample size [12]. Based on these parameters, the minimum required sample size was 385 participants. A convenience sampling technique was employed, whereby the questionnaire link was distributed electronically, and participation was voluntary.

Assessment Tool

The questionnaire was adapted from the tool developed by Geddawy et al., with permission obtained from the authors [5]. It consisted of 31 items. The instrument covered demographic characteristics, previous exposure to anesthesia, types and indications of anesthesia received, knowledge and awareness of general, regional, and local anesthesia, perceptions of the anesthesiologist’s roles and responsibilities, fears and concerns related to anesthesia, awareness of potential complications, informed consent, patient-anesthesiologist communication, trust and confidence in anesthesiologists, and satisfaction with the information provided. Responses were recorded using a Likert-scale format. Content validity of the questionnaire was assessed through review by two medical specialists. Internal consistency reliability was evaluated using Cronbach’s alpha, with a value of 0.70 or higher considered acceptable.

Participants' knowledge and awareness regarding anesthesia were assessed using a structured questionnaire with dichotomous and multiple-choice items (Appendices A-F). For dichotomous (yes/no) questions, responses were coded binarily, with *yes* assigned a value of 1 and *no* assigned 0. For items assessing familiarity or awareness levels with multiple response options, participants demonstrating awareness (responses indicating *somewhat aware*, *familiar*, or higher levels of knowledge) were coded as 1, while those indicating no awareness were coded as 0. This approach was adopted to capture clinically relevant awareness, as partial familiarity is sufficient to influence patient confidence and perioperative communication.

For multiple-choice questions requiring identification of correct answers, a response was scored as 1 if at least two correct options were selected, and 0 otherwise. Individual item scores were summed to generate a raw knowledge score, which was subsequently converted to a standardized scale ranging from 0 to 100, with higher scores indicating greater knowledge and awareness of anesthesia-related concepts. Perceptions toward anesthesia were evaluated using Likert-scale items. Responses reflecting positive perceptions were assigned higher numerical values according to the standard Likert scaling convention. Individual item scores were aggregated and transformed into a composite perception score on a scale of 0 to 100, where higher scores represented more favorable attitudes and perceptions toward anesthesia.

Ethical considerations

The study was conducted after obtaining approval from the Institutional Review Board of Dar Al Uloom University. All procedures adhered to the principles of the Declaration of Helsinki. An informed consent statement was presented at the beginning of the questionnaire, and only participants who provided consent were permitted to complete the survey. No personally identifiable information was collected, ensuring participant anonymity and confidentiality. Furthermore, strict measures were taken to ensure transparency, confidentiality, and data protection.

Statistical analysis

All statistical analyses were conducted using IBM SPSS Statistics version 30.0 (IBM Corp., Armonk, NY). Descriptive statistics, including frequencies and percentages, were computed for categorical variables, while means and standard deviations were calculated for continuous variables. Due to the non-normal distribution of awareness and perception scores, non-parametric tests were employed for group comparisons. Although awareness and perception scores demonstrated non-normal distributions, multiple linear regression was applied as these outcomes were continuous variables standardized on a 0-100 scale. The Mann-Whitney U test was used to compare scores between two independent groups (gender and age categories), while the Kruskal-Wallis H test was applied for comparisons across multiple groups (education level, previous anesthesia exposure, type of anesthesia administered, informed consent status, and preference for meeting the anesthesiologist). To identify the independent predictors of awareness and perception scores while controlling for potential confounding variables, multiple linear regression analyses were performed using the enter method. All statistical tests were two-tailed, and a *P*-value of less than 0.05 was considered statistically significant.

## Results

Out of 390 responses, 298 participants (76.4%) were included in the analysis. Excluded responses were from participants who were either younger than 18 years or did not receive anesthesia. Females comprised 52% (n=155) of the study sample. Education level showed that the majority completed high school (181, 60.7%), followed by post-graduate education (96, 32.2%), while only 21 (7.0%) had no formal schooling. General anesthesia was the most frequently administered type (190, 63.8%), followed by regional anesthesia (56, 18.8%) and local anesthesia (44, 14.8%), with only 8 (2.7%) unable to recall the type received. Regarding the decision-making process for anesthesia type, the majority reported it was the doctor's decision (178, 59.7%), while 59 (19.8%) made the decision based on their doctor's advice. A substantial proportion of participants were informed about consent for anesthesia (208, 69.8%), though 42 (14.1%) reported not being informed at all. When asked about preferences for preoperative consultation, 208 (69.8%) expressed a desire to meet the anesthesiologist before surgery. The most prevalent fear related to anesthesia was feeling pain during the procedure (138, 46.3%), followed by not waking up (75, 25.2%) and becoming unconscious (70, 23.5%) (Table 1).

**Table 1:** Demographic characteristics and previous anesthesia experience of participants (N = 298). Values are given as *n* (%).

Variable	*n* (%)
Gender	
Female	155 (52.0)
Male	143 (48.0)
Education level	
No school	21 (7.0)
High school	181 (60.7)
Post-graduate	96 (32.2)
Type of anesthesia administered	
General anesthesia	190 (63.8)
Local anesthesia	44 (14.8)
Regional anesthesia	56 (18.8)
I don’t know	8 (2.7)
Reason for anesthesia type	
Doctor’s decision	178 (59.7)
My decision based on the doctor’s advice	59 (19.8)
My decision alone	25 (8.4)
I don’t know	36 (12.1)
Informed about consent for anesthesia	
No	42 (14.1)
Yes	208 (69.8)
Only for surgeries	18 (6.0)
I don’t know	30 (10.1)
Preference for meeting the anesthesiologist before surgery	
No	37 (12.4)
Yes	208 (69.8)
Only surgeon	39 (13.1)
Both the surgeon and the anesthesiologist	14 (4.7)
Fears related to anesthesia	
Becoming unconscious	70 (23.5)
Not waking up	75 (25.2)
Feeling pain	138 (46.3)
Unable to move	9 (3.0)
I don’t know	6 (2.0)

Knowledge and awareness about different types of anesthesia and the anesthesiologist's role demonstrated considerable variation among participants. The majority reported knowledge about general anesthesia (272, 91.3%) and local anesthesia (263, 88.3%), while awareness of regional anesthesia was moderate (186, 62.4%). However, knowledge of more specialized techniques remained limited, with only 47 (15.8%) familiar with spinal anesthesia, 39 (13.1%) with epidural anesthesia, and 45 (15.1%) with peripheral neuro-axial blocks. Familiarity with the anesthesiologist's role was generally positive, as 200 (67.1%) reported being somewhat familiar and 39 (13.1%) were very familiar, while only 51 (17.1%) were not familiar and 8 (2.7%) had never heard of it. Regarding awareness of complications associated with regional anesthesia, 147 (49.3%) identified muscle weakness as a potential complication, followed by nerve damage (55, 18.5%) and back pain (48, 16.1%), though 48 (16.1%) did not know complications. Understanding of the anesthesiologist's intraoperative responsibilities was strong, with 71.1% (212) correctly identifying that the anesthesiologist remains in the operating room throughout the procedure. When asked who would provide resuscitation during a crisis, 158 (53.0%) correctly identified the anesthesiologist, while 90 (30.2%) indicated that all team members would be involved. The vast majority of participants, 231 (77.5%), correctly recognized that the anesthesiologist is responsible for patient recovery, and 179 (60.1%) reported having a general idea of the anesthesiologist's expertise, with an additional 81 (27.2%) claiming to be fully aware (Table 2).

**Table 2:** Knowledge and awareness about anesthesia and the anesthesiologist’s role (N = 298). Values are given as *n* (%).

Variable	*n* (%)
Knowledge about general anesthesia	
No	26 (8.7)
Yes	272 (91.3)
Knowledge about regional anesthesia	
No	112 (37.6)
Yes	186 (62.4)
Knowledge about local anesthesia	
No	35 (11.7)
Yes	263 (88.3)
Knowledge about spinal anesthesia	
No	251 (84.2)
Yes	47 (15.8)
Knowledge about epidural anesthesia	
No	259 (86.9)
Yes	39 (13.1)
Knowledge about the peripheral neuro-axial block	
No	253 (84.9)
Yes	45 (15.1)
Familiarity with the anesthesiologist’s role	
Never heard of it	8 (2.7)
Not familiar	51 (17.1)
Somewhat familiar	200 (67.1)
Very familiar	39 (13.1)
Awareness of complications of regional anesthesia	
Nerve damage	55 (18.5)
Muscle weakness	147 (49.3)
Back pain	48 (16.1)
No idea	48 (16.1)
Anesthesiologist remains in the room throughout the procedure	
No	26 (8.7)
Yes	212 (71.1)
Maybe	51 (17.1)
I don’t know	9 (3.0)
Who would resuscitate during a crisis	
Anesthesiologist	158 (53.0)
Surgeon	12 (4.0)
Nurse	11 (3.7)
Technician	27 (9.1)
All	90 (30.2)
Anesthesiologist responsible for patient recovery	
No	32 (10.7)
Yes	231 (77.5)
I don’t know	35 (11.7)
Knowledge of an anesthesiologist’s expertise	
No	29 (9.7)
Yes, I have a general idea	179 (60.1)
Yes, I am fully aware	81 (27.2)
I never thought about it	9 (3.0)

Patient satisfaction and trust in anesthesiologists demonstrated predominantly positive trends, with notable improvements in communication quality compared to baseline expectations. When asked about explanations of anesthetic processes and risks, 106 (35.6%) reported receiving complete explanations, 158 (53.0%) received partial explanations, and 34 (11.4%) received no explanation. Similarly, regarding addressing patient concerns and questions, 73 (24.5%) reported that their queries were thoroughly addressed, 192 (64.4%) received partial responses, and 22 (7.4%) felt their concerns were not addressed. Overall satisfaction with the information provided was high, with 192 (64.4%) reporting satisfaction and 76 (25.5%) reporting being very satisfied, while 18 (6.0%) remained neutral and 12 (4.0%) were dissatisfied. Communication skills of anesthesiologists were rated favorably, with 166 (55.7%) rating them as excellent and 99 (33.2%) as good, while 18 (6.0%) rated them as fair and 15 (5.0%) as poor. Participants' willingness to openly express concerns was moderate to high, with 56 (18.8%) always feeling comfortable, 178 (59.7%) sometimes feeling comfortable, 53 (17.8%) rarely feeling comfortable, and 11 (3.7%) never feeling comfortable. Recognition of the anesthesiologist's importance in ensuring safe surgery was substantial, with 157 (52.7%) considering it extremely important and 86 (28.9%) moderately important. Trust in anesthesiologists for pain and comfort management was high, with 84 (28.2%) reporting full trust and 187 (62.8%) reporting some trust. Confidence in the anesthesiologist's ability to handle complications was similarly high, with 89 (29.9%) reporting being very confident and 189 (63.4%) somewhat confident. The anesthesiologist's experience greatly affected patient confidence for 78 (26.2%) of participants and moderately affected 191 (64.1%), while the hospital's reputation moderately affected confidence for 187 (62.8%) of participants (Table 3).

**Table 3:** Patient-anesthesiologist communication, satisfaction, and trust (N = 298). Values are presented as *n* (%).

Variable	*n* (%)
Anesthesiologist explained the anesthesia process and risks	
No	34 (11.4)
Yes, partially	158 (53.0)
Yes, completely	106 (35.6)
Anesthesiologist addressed concerns and questions	
No	22 (7.4)
Yes, partially	192 (64.4)
Yes, thoroughly	73 (24.5)
I did not have any queries	11 (3.7)
Satisfaction with information provided	
Dissatisfied	12 (4.0)
Neutral	18 (6.0)
Satisfied	192 (64.4)
Very satisfied	76 (25.5)
Rating of an anesthesiologist’s communication skills	
Poor	15 (5.0)
Fair	18 (6.0)
Good	99 (33.2)
Excellent	166 (55.7)
Openness in expressing concerns to an anesthesiologist	
Never	11 (3.7)
Rarely	53 (17.8)
Sometimes	178 (59.7)
Always	56 (18.8)
Perceived importance of an anesthesiologist’s role in safe surgery	
Not important	31 (10.4)
Slightly important	24 (8.1)
Moderately important	86 (28.9)
Extremely important	157 (52.7)
Trust in an anesthesiologist for pain and comfort management	
Do not trust	7 (2.3)
Neutral	20 (6.7)
Somewhat trust	187 (62.8)
Fully trust	84 (28.2)
Confidence in an anesthesiologist’s ability to handle intraoperative complications	
Not confident	3 (1.0)
Neutral	17 (5.7)
Somewhat confident	189 (63.4)
Very confident	89 (29.9)
Effect of an anesthesiologist’s experience on patient confidence	
Does not affect	7 (2.3)
Slightly affects	22 (7.4)
Moderately affects	191 (64.1)
Greatly affects	78 (26.2)
Effect of the hospital’s reputation on confidence in an anesthesiologist	
Does not affect	2 (0.7)
Slightly affects	58 (19.5)
Moderately affects	187 (62.8)
Greatly affects	51 (17.1)

Comparative analysis of awareness and perception scores across demographic and clinical variables revealed significant differences across multiple factors. Female participants demonstrated significantly higher awareness scores (*M* = 65.8, standard deviation (SD) = 7.1) compared to males (*M* = 58.6, SD = 13.4, *P* < 0.001), though perception scores did not differ significantly between genders (*P* = 0.676). Educational level significantly influenced awareness scores (*P* < 0.001), with high school-educated participants showing the highest awareness (*M* = 65.8, SD = 6.5), followed by those with no formal schooling (*M* = 62.3, SD = 19.5) and post-graduate education (*M* = 55.8, SD = 12.7). Education did not significantly affect perception scores (*P* = 0.084). Type of anesthesia administered did not significantly affect awareness scores (*P* = 0.187), but significantly influenced perception scores (*P* < 0.001), with participants who received general anesthesia demonstrating the highest perception scores (*M* = 79.0, SD = 5.4), while those who received local anesthesia (*M* = 74.2, SD = 7.6) or regional anesthesia (*M* = 74.2, SD = 8.2) had lower scores. Being informed about consent for anesthesia was associated with significantly higher awareness (*M* = 65.1, SD = 7.1) and perception (*M* = 79.1, SD = 5.4) scores compared to those not informed (both *P* < 0.001). Preference for meeting the anesthesiologist before surgery showed significant associations with both outcomes (both *P* < 0.001), with participants expressing a desire to meet the anesthesiologist having the highest awareness (*M* = 65.7, SD = 8.4) and perception (*M* = 79.4, SD = 5.1) scores, while those preferring to meet only the surgeon had the lowest awareness scores (*M* = 49.1, SD = 12.2) (Table 4).

**Table 4:** Comparison of awareness and perception scores across demographic and clinical variables (N = 298). Mann-Whitney U test for dichotomous variables; Kruskal-Wallis H test for variables with more than two categories.

Variable	Category	Awareness score, mean (SD)	Test statistics (*P*-value)	Perception score, mean (SD)	Test statistics (*P*-value)
Gender	Female	65.8 (7.1)	Mann-Whitney U = 7050 (<0.001)	77.4 (6.0)	Mann-Whitney U = 10777.5 (0.676)
	Male	58.6 (13.4)		76.9 (7.8)	
Education level	No school	62.3 (19.5)	Kruskal-Wallis *H* = 64.934 (<0.001)	74.1 (10.3)	Kruskal-Wallis *H* = 4.947 (0.084)
	High school	65.8 (6.5)		77.3 (5.9)	
	Post-graduate	55.8 (12.7)		77.5 (7.7)	
Type of anesthesia administered	General anesthesia	62.1 (9.7)	Kruskal-Wallis *H* = 4.806 (0.187)	79.0 (5.4)	Kruskal-Wallis *H* = 28.297 (<0.001)
	Local anesthesia	62.5 (13.6)		74.2 (7.6)	
	Regional anesthesia	64.6 (12.0)		74.2 (8.2)	
	I don’t know	52.1 (17.1)		71.6 (10.8)	
Informed about consent	No	59.3 (14.3)	Kruskal-Wallis *H* = 59.340 (<0.001)	72.9 (9.6)	Kruskal-Wallis *H* = 50.734 (<0.001)
	Yes	65.1 (7.1)		79.1 (5.4)	
	Only for surgeries	63.4 (17.4)		70.0 (7.2)	
	I don’t know	46.9 (11.9)		74.4 (5.3)	
Preference for meeting anesthesiologist	No	59.9 (11.3)	Kruskal-Wallis *H* = 72.533 (<0.001)	73.7 (9.2)	Kruskal-Wallis *H* = 73.219 (<0.001)
	Yes	65.7 (8.4)		79.4 (5.1)	
	Only surgeon	49.1 (12.2)		71.9 (6.3)	
	Both surgeon and anesthesiologist	55.4 (12.9)		68.7 (7.4)	

Multiple regression analysis predicting awareness scores revealed a significant overall model (*R*² = 0.406, adjusted *R*² = 0.381, *F*(12, 285) = 16.210, *P* < 0.001), accounting for 40.6% of the variance in awareness. Receiving regional anesthesia positively predicted awareness (*B* = 4.03, 95% CI 1.29-6.76, β = 0.141, *P* = 0.004), as did being informed about consent only for surgeries (*B* = 6.97, 95% CI 1.86-12.08, β = 0.149, *P* = 0.008). Preference to meet the anesthesiologist was a positive predictor (*B* = 4.22, 95% CI 0.78-7.66, β = 0.174, *P* = 0.016), while preference to meet only the surgeon was a strong negative predictor (*B* = -7.64, 95% CI -12.13 to -3.14, β = -0.231, *P* < 0.001). Post-graduate education approached but reached statistical significance as a negative predictor (*B* = -4.45, 95% CI -8.90 to -0.00, β = -0.187, *P* = 0.050) (Table 5).

**Table 5:** Multiple regression analysis predicting awareness scores (N = 298). Note: *R*² = 0.406, adjusted *R*² = 0.381, *F*(12, 285) = 16.210, *P* < 0.001. Reference categories: gender (female), education (no schooling), type of anesthesia (general anesthesia), informed consent (no), and preference to meet (no).

Predictor	*B* (95% CI)	β	*P*-value
Gender (Male)	-1.07 (-3.55 to 1.42)	-0.048	0.399
Education level: High school	1.02 (-3.34 to 5.38)	0.045	0.646
Education level: Post-graduate	-4.45 (-8.90 to -0.00)	-0.187	0.050
Type of anesthesia: Local anesthesia	1.37 (-1.90 to 4.64)	0.044	0.409
Type of anesthesia: Regional anesthesia	4.03 (1.29-6.76)	0.141	0.004
Type of anesthesia: I don’t know	-3.34 (-9.88 to 3.21)	-0.048	0.316
Informed about consent: Yes	1.30 (-2.01 to 4.62)	0.054	0.439
Informed about consent: Only for surgeries	6.97 (1.86-12.08)	0.149	0.008
Informed about consent: I don’t know	-4.36 (-9.16 to 0.45)	-0.118	0.075
Preference to meet an anesthesiologist: Yes	4.22 (0.78 to 7.66)	0.174	0.016
Preference to meet an anesthesiologist: Only the surgeon	-7.64 (-12.13 to -3.14)	-0.231	<0.001
Preference to meet an anesthesiologist: Both surgeon and anesthesiologist	-4.52 (-10.13 to 1.08)	-0.086	0.113

Multiple regression analysis for perception scores yielded a significant model (*R*² = 0.374, adjusted *R*² = 0.347, *F*(12, 285) = 14.165, *P* < 0.001), explaining 37.4% of the variance. Being informed about consent for anesthesia was a positive predictor (*B* = 3.10, 95% CI 0.99-5.20, β = 0.206, *P* = 0.004), as was preference to meet the anesthesiologist (*B* = 4.51, 95% CI 2.32-6.70, β = 0.300, *P* < 0.001), which emerged as the strongest predictor. Receiving regional anesthesia negatively predicted perception scores (*B* = -3.61, 95% CI -5.35 to -1.87, β = -0.204, *P* < 0.001), as did preference to meet both surgeon and anesthesiologist (*B* = -4.07, 95% CI - 7.63 to -0.50, β = -0.125, *P* = 0.026) (Table 6).

**Table 6:** Multiple regression analysis predicting perception scores (N = 298). Note: *R*² = 0.374, adjusted *R*² = 0.347, *F*(12, 285) = 14.165, *P* < 0.001. Reference categories: gender (female), education (no schooling), type of anesthesia (general anesthesia), informed consent (no), and preference to meet (no).

Predictor	*B* (95% CI)	β	*P*-value
Gender (Male)	0.06 (-1.53 to 1.64)	0.004	0.946
Education level: High school	-2.02 (-4.79 to 0.76)	-0.143	0.154
Education level: Post-graduate	1.62 (-1.21 to 4.45)	0.109	0.262
Type of anesthesia: Local anesthesia	-1.93 (-4.01 to 0.15)	-0.099	0.069
Type of anesthesia: Regional anesthesia	-3.61 (-5.35 to -1.87)	-0.204	<0.001
Type of anesthesia: I don’t know	-2.69 (-6.86 to 1.47)	-0.063	0.204
Informed about consent: Yes	3.10 (0.99-5.20)	0.206	0.004
Informed about consent: Only for surgeries	-2.13 (-5.38 to 1.12)	-0.074	0.197
Informed about consent: I don’t know	1.28 (-1.78 to 4.34)	0.056	0.411
Preference to meet an anesthesiologist: Yes	4.51 (2.32-6.70)	0.300	<0.001
Preference to meet an anesthesiologist: Only the surgeon	-2.09 (-4.95 to 0.77)	-0.102	0.151
Preference to meet an anesthesiologist: Both surgeon and anesthesiologist	-4.07 (-7.63 to -0.50)	-0.125	0.026

## Discussion

The findings of the present study showed that while basic awareness of anesthesia is relatively high, detailed understanding remains limited. The vast majority had heard of general (91.3%) and local (88.3%) anesthesia, but only a few participants knew about spinal or epidural techniques. This aligns with findings from previous studies. For example, Arefayne et al., in their study involving 307 patients, reported that most participants demonstrated poor knowledge of anesthesia and the role of anesthetists, with 220 individuals (71.7%) answering fewer than half of the knowledge questions correctly. Only 87 participants (28.3%) showed a good level of knowledge. Previous exposure to anesthesia (*P* = 0.001) and occupation (*P* = 0.022) were significantly associated with higher knowledge levels [4]. Similarly, a cross-sectional study involving 411 perioperative patients reported that overall knowledge score regarding anesthesiologists’ roles was low (59.4% ± 18.8), with most patients unaware of anesthesiologists’ responsibilities outside the operating room. Knowledge scores were significantly higher among patients with university education and those from medical or health-related fields (both *P* < 0.001), and showed a weak positive correlation with self-rated general health (*r* = 0.17, *P* < 0.001) [7].

Patient characteristics also influenced the awareness level. Female participants had significantly higher awareness scores than males. Similar findings have been reported previously. Vahabi et al., in their study, reported that women were more aware of general and local anesthesia compared to men [13]. However, several other studies have not found a significant association between gender and awareness level about anesthesia [14,15]. For example, Wu et al. did not find any significant difference between males and females regarding knowledge (*P* = 0.18) and attitude (*P* = 0.77) towards anesthesia [14]. Similarly, Zahran et al. reported no significant difference between males and females and the knowledge level of anesthesia (*P* = 0.62) [7]. However, Fentie and Simegnew reported that males were 1.9 times more aware of anesthesia compared to females (*P *= 0.04) [16]. Regarding education level, only those with a high school education scored higher compared to those with post-graduate education. This contrasts with many reports that higher formal education generally predicts better medical knowledge. For example, Zahran et al. found university-educated patients had significantly higher anesthesia knowledge [7]. Similarly, Djagbletey et al., in their study, reported that those with tertiary education had significantly higher knowledge levels compared to those with no or basic education (*P* = 0.00) [17]. Similar findings have been reported by other studies [14,16]. Several factors may help explain this observation. First, the postgraduate group in this study may have consisted largely of individuals from non-health-related disciplines, whose advanced education does not necessarily translate into greater exposure to medical concepts. Second, highly educated individuals may overestimate their understanding of anesthesia and therefore engage less actively in preoperative discussions, potentially leading to gaps in retained knowledge.

Prior anesthesia experience and exposure also played a role. We did not find a significant difference in awareness by type of anesthesia received, but patients who had general anesthesia scored highest on perception. Similar to our findings, Djagbletey et al. also did not find any significant difference regarding knowledge level about anesthesia and previous anesthesia or surgery exposure [17]. Similarly, Wu et al. found that patients undergoing general anesthesia had better knowledge compared to other groups, and this difference was also statistically significant, which differs from our study. However, Vahabi et al. reported that patients with previous local anesthesia or surgeries tended to have distinct knowledge levels about anesthesia [13]. Another important finding of our study is that being informed about anesthesia consent was strongly associated with better outcomes. Participants who recalled receiving a consent discussion had significantly higher awareness and perception scores (*P *< 0.001 for both). This point highlights that structured preoperative education regarding anesthesia is effective for patients. Our findings align with Jaju et al., who also advocated that prioritizing effective communication and shared decision-making during pre-anesthesia visits significantly enhances patient comprehension and satisfaction [18]. Patients in our study reported common anesthesia-related concerns. The most frequent fear was intraoperative pain (46.3%), followed by not waking up (25.2%) and losing consciousness (23.5%). These anxieties are consistent with previous studies. Jovanovic et al. reported that many surgical patients fear anesthesia complications and not waking up, with women and those with less knowledge more likely to have these fears [19].

Similar to the present study, D'Agostino et al. also reported that 34.6% patients in their study feared not waking up from anesthesia, followed by pain during surgery (25.2%) [20]. In our cohort, 59% sometimes and 18.8% always felt comfortable voicing concerns to the anesthesiologist, whereas only 3.7% never did. This relatively high openness reflects trust in the care team. The literature shows that patient fears can negatively impact perioperative anxiety and outcomes, and that education helps mitigate this [19]. Awareness of anesthetic complications was limited. About half knew that muscle weakness can follow regional anesthesia, but many were unaware of potential nerve damage or back pain, and 16% had no idea of any complications. Similarly, misconceptions about anesthesiologists’ roles were also reported as 53% correctly identifying the anesthesiologist as the person who would resuscitate during a crisis, but 30% thought all team members would share that responsibility. These findings align with a previous study by Bazaid et al. from Saudi Arabia, who reported that 82% correctly recognized that anesthesiologists administer anesthesia, yet many still held inaccurate beliefs about perioperative responsibilities [21]. In the present study, patient satisfaction with anesthesiologist communication was very high. Over 88.9% rated communication skills as good or excellent, and 89.9% were satisfied or very satisfied with the information provided. Such positive feedback is in line with the principle that good communication boosts satisfaction. For example, Salgaonkar et al., in their study involving anesthesiology residents, reported that all residents felt that good preoperative communication can improve patient satisfaction [22].

Our regression analysis identified key modifiable predictors. The strongest positive predictor for both awareness and perception scores was expressing a preference to meet the anesthesiologist before surgery. This suggests patient engagement with the anesthesiology team may itself improve patient understanding and satisfaction.

Limitations

This study has certain limitations as well, which should be considered while interpreting the findings. The use of convenience sampling and an online questionnaire distributed via Google Forms may have introduced selection bias. Individuals with higher educational levels, greater digital access, or increased interest in health-related topics may have been more likely to participate. The final sample size (*n* = 298) was lower than the calculated target (*n* = 385), which may have reduced statistical power, particularly in smaller subgroups such as regional anesthesia. Data were collected using a self-reported online questionnaire, introducing potential selection and recall bias, and anesthesia-related details were not verified against medical records. Important variables such as time since last anesthesia exposure and detailed age categories were not captured, limiting adjustment for confounding factors. The cross-sectional design captures associations but not causality. Our setting in Saudi Arabia may limit generalizability to other regions or cultures.

## Conclusions

This study revealed important insights into public awareness, knowledge, and perceptions regarding anesthesia and the anesthesiologist's role. While the majority of participants demonstrated strong familiarity with general and local anesthesia, knowledge of regional anesthetic techniques such as spinal, epidural, and peripheral nerve blocks remained notably limited, with fewer participants reporting awareness of these specialized modalities. This knowledge gap represents a significant opportunity for enhanced patient education initiatives. Communication between anesthesiologists and patients emerged as a strength in this cohort. Patient satisfaction, trust, and confidence levels were predominantly high, with over 90% of participants expressing at least some trust in anesthesiologists for pain management and confidence in their ability to handle complications. Multiple regression analyses identified several key predictors of awareness and perception scores. Preference to meet the anesthesiologist before surgery emerged as the strongest predictor of perception scores and a significant positive predictor of awareness scores, highlighting the critical importance of preoperative anesthetic consultation in enhancing patient understanding and satisfaction. Gender differences were notable, with female participants showing significantly higher awareness scores than males. In clinical practice, this can be operationalized through structured preoperative anesthesia consultations, including standardized counseling protocols, dedicated pre-anesthesia assessment clinics, and the use of clear, patient-friendly educational materials addressing anesthesia types, risks, and expectations. However, these findings need to be validated in much larger cohorts.

## Figures and Tables

**Figure 1:**
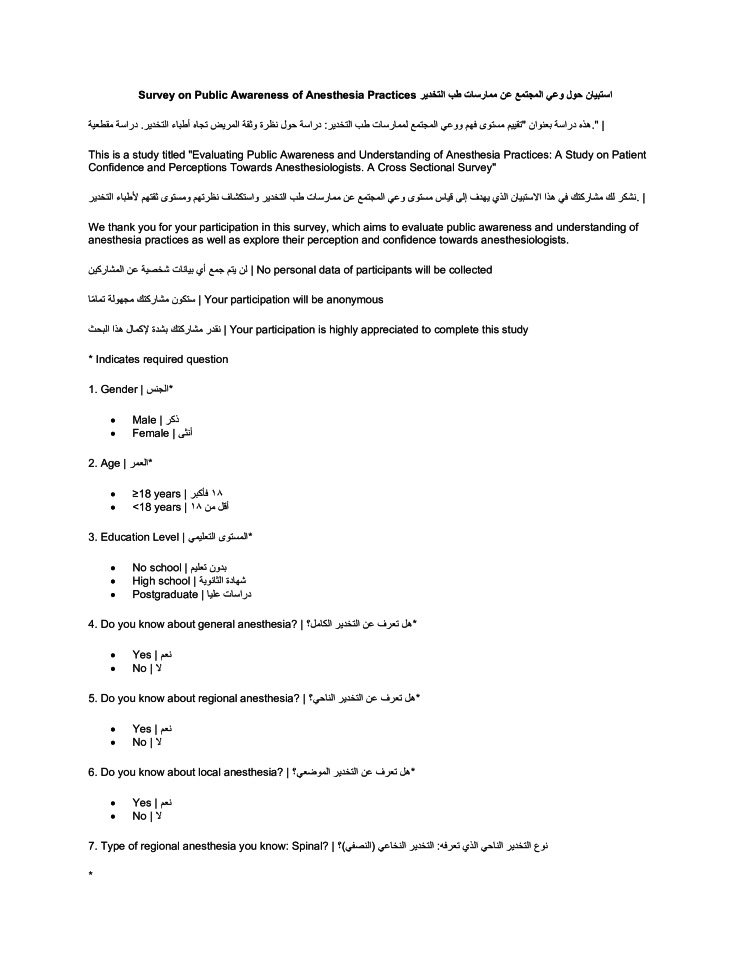
Survey on public awareness of anesthesia practices (Questions 1-7). These are the parts of the survey questionnaire.

**Figure 2:**
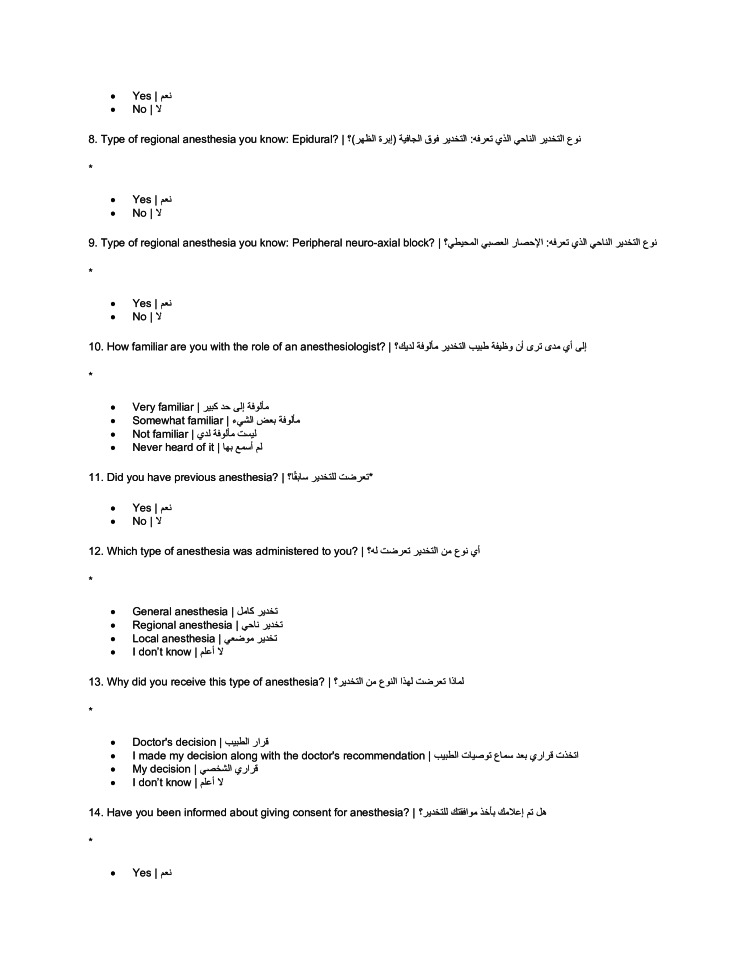
Survey on public awareness of anesthesia practices (Questions 8-14). These are the parts of the survey questionnaire.

**Figure 3:**
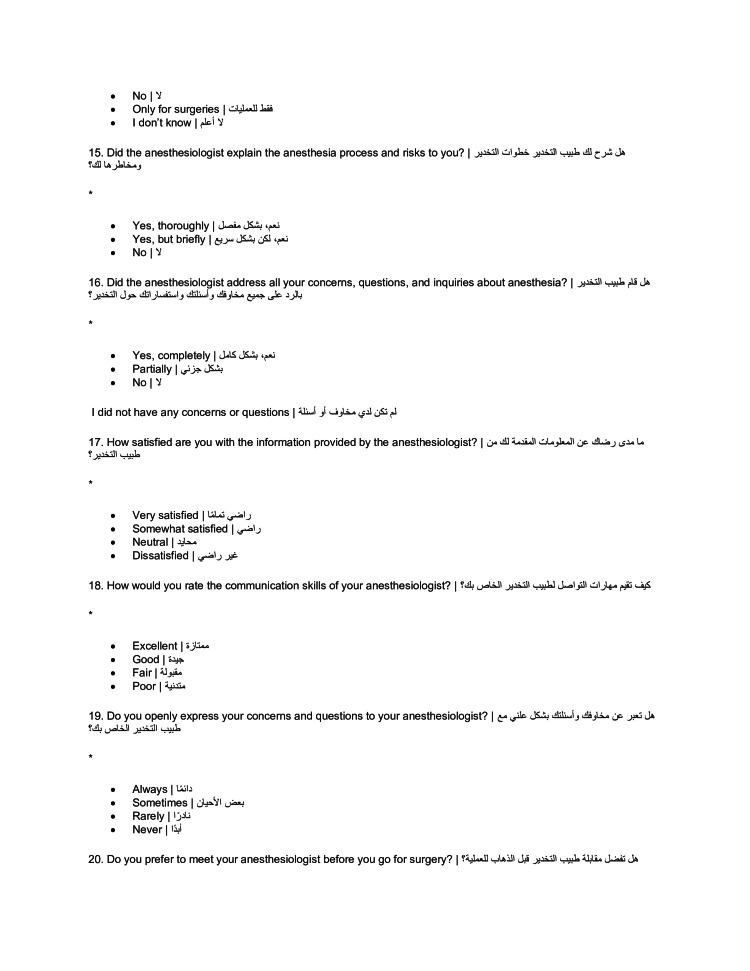
Survey on public awareness of anesthesia practices (Questions 15-20). These are the parts of the survey questionnaire.

**Figure 4:**
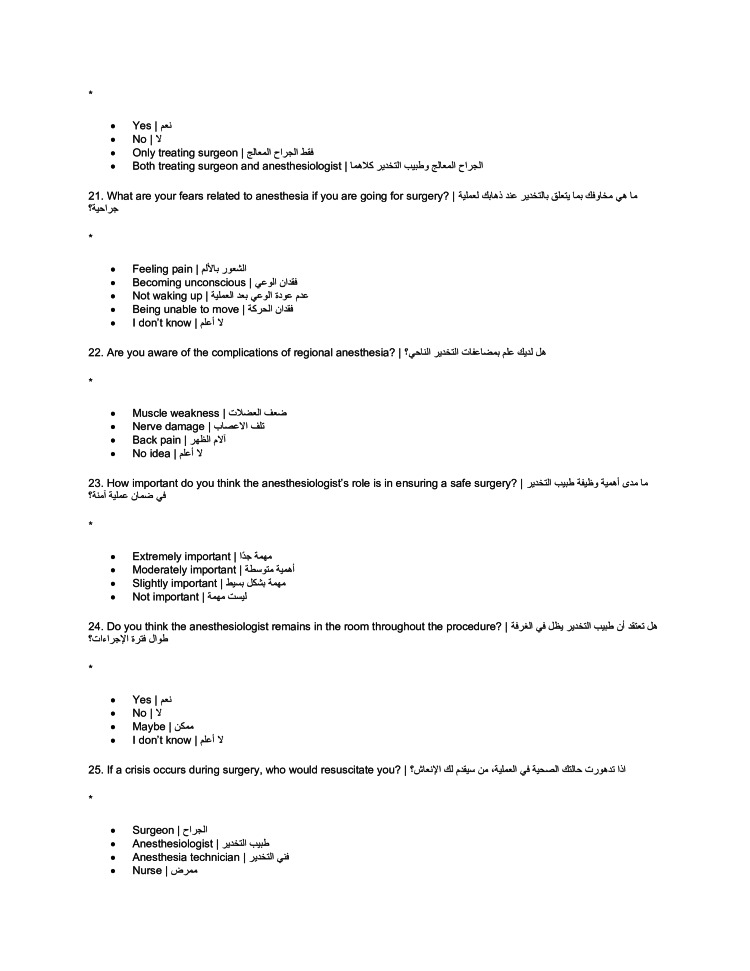
Survey on public awareness of anesthesia practices (Questions 21-25). These are the parts of the survey questionnaire.

**Figure 5:**
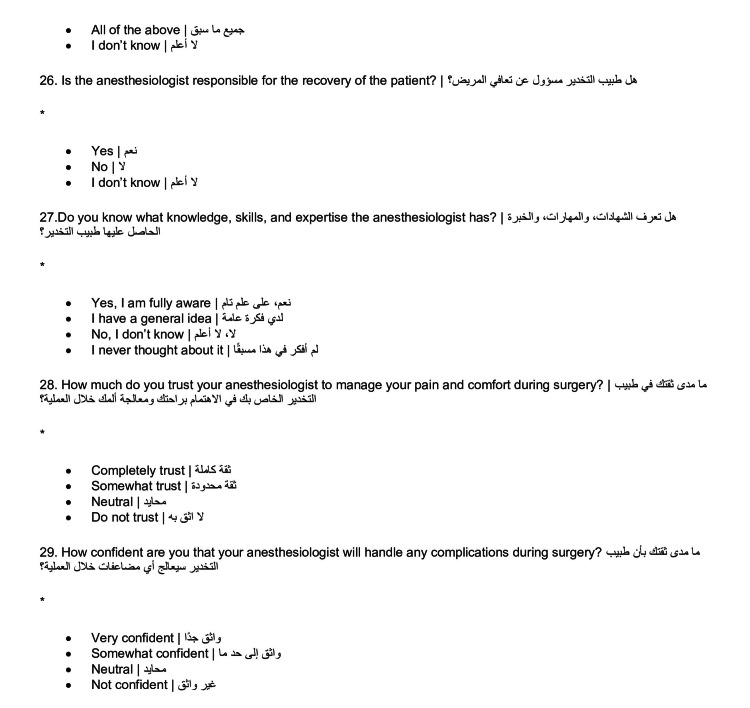
Survey on public awareness of anesthesia practices (Questions 26-29). These are the parts of the survey questionnaire.

**Figure 6:**
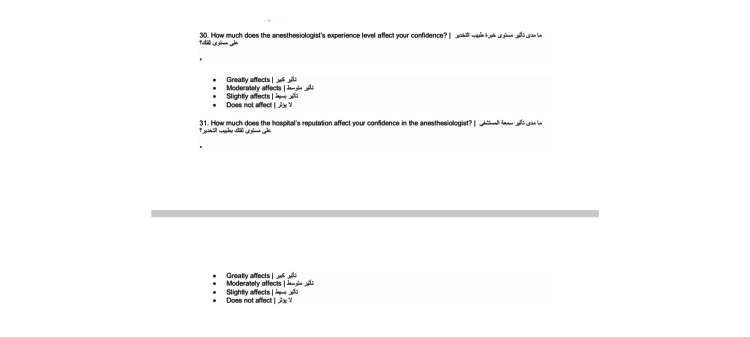
Survey on public awareness of anesthesia practices (Questions 30-31). These are the parts of the survey questionnaire.

## Appendices

Appendix A

Appendix B

Appendix C

Appendix D

Appendix E

Appendix F
